# How is Participating in Suicide Prevention Activities Experienced by Those with Lived and Living Experiences of Suicide in Australia? A Qualitative Study

**DOI:** 10.3390/ijerph17134635

**Published:** 2020-06-27

**Authors:** Sarah Wayland, Kathy McKay, Myfanwy Maple

**Affiliations:** 1 School of Health, Faculty of Medicine and Health, University of New England, Armidale 2351, Australia; mmaple2@une.edu.au; 2 Sydney School of Health Sciences, Faculty of Medicine and Health, University of Sydney, Camperdown 2050, Australia; 3 Public Health, Policy and Systems, Institute of Population Health, University of Liverpool, Liverpool L69 3GL, UK; KMcKay@Tavi-Port.nhs.uk; 4 Tavistock and Portman NHS (National Health Service) Foundation Trust, London NW3 5BA, UK

**Keywords:** suicide, suicide prevention, suicide postvention, lived experience, collaboration, participation

## Abstract

People with a lived experience of suicide are commonly included within suicide prevention research. This includes participation in conferences, policy development, research and other activities. Yet little is known about the impact on the person in the long term of regularly sharing one’s experience to different audiences and, in some cases, to a schedule not of your choosing. This qualitative study asked twenty people to share their reflections of being lived experience representatives within suicide prevention. Participants varied in the length of time they had been sharing their stories, and how they shared with different audiences. These narratives were thematically analysed within a reflective framework, including field notes. Four broad themes were noted that highlighted participants’ recommendations as to how the lived experience speaker training could grow alongside suicide prevention activities to facilitate safe activities that include a shared understanding of the expected outcome from participation. The environment for people with lived experience of suicide to tell their stories already exists, meaning that the suicide prevention sector needs to move quickly to ensure people understand the variety of spaces where lived experience needs to be incorporated, evaluated and better supported. When lived experience is a valued inclusion in the creation of effective and appropriate suicide prevention research and interventions, those who share their experience must be valued and supported in a way that reflects this. This study recommends strategies to practically and emotionally support speakers, including ways to ensure debriefing and support, which can enhance the longevity of the speakers in the suicide prevention space by valuing the practical and emotional labour required to be suicide prevention representatives, with an outcome recommendation for best practice guidelines for those who engage people with lived experience in suicide prevention activities.

## 1. Introduction

Grounding research in the first-hand accounts of people who have lived through, or continue to live with, significant and ongoing life events has become prominent in the health and social care sector, particularly in the field of disability, mental health/illness and AIDS (Acquired Immunodeficiency Syndrome) [1,2]. In relation to research activities incorporating first-hand accounts, there has been significant work investigating the most appropriate methodologies to be used in these studies, especially when the lived experience is a sensitive one [3] or when children are involved [4]. Much of this work has privileged the stories of people as vital to understanding not only how people experience health or illness, but how they navigate health and social systems and the different treatments and support offered during this process [5]. In the Australian context, active participation, where people share about the impacts of their life experiences, has long shaped programs and services in the health system [6]. Lived experience can both demonstrate why an intervention may or may not be effective or appropriate, as well as how that intervention works in everyday life [7]. Recently, this same movement has emerged in the suicide prevention sector, with the inclusion of lived experience now viewed as integral to most suicide prevention activities in Australia. Examples of how this has quickly become embedded in suicide prevention include, for example, the New South Wales Mental Health Commission’s Lived Experience Framework that seeks to lead and influence the mental health system [8].

In Australia, the term “lived experience of suicide” broadly refers to those who have survived their own suicide attempt, been bereaved by the death of someone who died by suicide or supported someone who was/is suicidal. Organisations such as Suicide Prevention Australia, Black Dog Institute and Beyond Blue have developed definitions, guidelines and principles for the inclusion of those with lived experience to ensure that the process is done safely [6]. Consequently, Speakers Bureaus and representative bodies have been established. The need for such formalisation has become apparent. Over the past decade, there has been exponential growth in the number of people talking publicly about their lived experiences of suicide, and the development of training to support those undertaking these speaking engagements [9]. Places are now included in every conference, meeting and other suicide prevention-related activity for people with lived experience. Personal stories are shared on research websites, social media, and blogs. Yet, thus far, the evidence base supporting the inclusion of these activities and how those involved experience undertaking these roles, including the impact of doing so, is not well developed. Further, there is also a dearth of research examining the impact that regularly sharing one’s lived experience of suicide has on the person over a long period of time. While the potentially cathartic nature of story-telling is well known [10], less well known is whether retelling a story that is traumatic, even if framed in terms of survival, on a regular basis to an audience and on a schedule not necessarily of your choosing, has the same positive impact. 

The review of the literature revealed no current evidence base to understand the experience of those who are undertaking suicide prevention lived experience speaking, nor how they perceive their impact of doing so. From a broad analysis of the suicide prevention literature, it was identified that “well-designed qualitative research adds significant context regarding the lived experience of those who are, or have been suicidal” [11]. Qualitative studies seeking to explore evaluation of the effectiveness of suicide prevention interventions have been minimal, as standalone methodologies. Humensky et al. [12] explored the reflections of the Life is Precious (LIP) program through focus groups with adolescent participants and mothers to learn whether participants and families believe that the activities of LIP address risks for suicidal behaviour. Skerett et al. [13] looked at the implications of a group-based model to engage people in speaking about suicidal behaviours. There has been no qualitative studies conducted to look at the role of speaking regularly about lived experience of suicide, as a strategy to offer others suicide prevention interventions. 

The aim of this project was to elucidate the narratives of lived experience representatives and advocates within the suicide prevention sector in Australia. Thus, a qualitative research project was designed to deeply explore the topic using a narrative inquiry methodology [14] to explore the research question: “How is authentic inclusion of lived experience, experienced by those who undertake suicide prevention activities in Australia?” This was designed to explore what it means to be a lived experience speaker or representative, what motivates people to become involved in these activities and how undertaking these events is experienced. People new to lived experience speaking, as well as those who regularly engage in speaking and those involved in the broader practices of policy and legislative development, were included to understand how identity, value and well-being are constructed and negotiated by the individuals as well as the people and organisations around them in this rapidly developing activity. 

## 2. Methods

The purpose of the qualitative research design was to allow participants to ‘sing up many truths/narratives’ [15] p.115 about their motivation for, and decision to, volunteer or work as lived experience speakers and representatives, and to identify the practical and emotional impacts of these activities. The research was approved by the University of New England Human research ethics committee (HE18-126, 1 June 2018).

To be eligible to participate, individuals were required to be 18 years of age and over, have a self-reported lived experience of suicide (their own prior suicide attempt, caring for someone who has made a suicide attempt or is suicidal, or bereaved by suicide), have previously undertaken speaker training (self-defined as having participated in training in how to tell of their lived experience), been engaged in voluntarily speaking about their lived experience for more than 12 months, and located in Australia. To ensure ongoing support for the person, a final inclusion criterion was an understanding of the support services provided to speakers. Those without lived experience of suicide, or who had not completed speaker training, or who had been engaged in speaking about their experience for less than 12 months, or were not located in Australia were excluded.

Purposive sampling to identify up to twenty participants was utilised to locate individuals willing to participate via organisations that provided speaker training. The sample size was pre-determined via Mason [16]. To contact potential participants, a participant information flyer was shared via Suicide Prevention Australia and Roses in the Ocean. These two organisations were identified as they do, or have previously, provided speaker training for this purpose in Australia. Further distribution of the information flyer was subsequently shared by recipients with additional speakers who had received training from other organisations thus identified. Participants were asked to contact the research team directly to ensure there was not any unintentional coercion by those who provide training to contact on behalf of the participant. The participant information sheet was sent via email, including the participant consent form. Verbal or written consent were received at the time of the interview depending on the interview being in person or via telephone. 

Given that the focus of this project was to highlight the inclusion of lived experience, lived experience inclusion was incorporated in all phases of this study. This study was partially funded by lived experience organisation, Roses in the Ocean. While this funding did not influence the outcomes presented, review of the interview schedule and publication were undertaken. Further one author on this paper has lived experience and was involved in the analysis of the interviews. Interviews were conducted between December 2018 and February 2019 using a semi-structured interview approach, to allow for co-construction of understanding within and between the interviews dependant on the narratives provided by participants. The interview guide is attached as Appendix A. To ensure academic rigour, all interviews were transcribed verbatim. Analysis of the transcripts was combined with the inclusion of the written field notes [17] from the two interviewers (S.W. and M.M.) taken during the data collection period, which lead to the identification of three topic areas (M.M. and S.W.). The transcripts were then reviewed independently and coded by a third researcher (K.M.) to identify block themes and persistent narratives. These were returned to S.W. and M.M., who then reviewed the emerging themes to verify their accuracy and identified subthemes. In addition, uncommon experiences were discussed across all authors to examine how these informed the emergent common themes. The focus of this process of thematic analysis was to group similar responses together, using systematic identifying, organising and grouping of patterns of meaning across the data, to gain greater understanding into the collective meanings and themes within [18] lived experience speaking. 

## 3. Findings

The findings demonstrated a broad range of experiences in both time since speaker training and active participation in the suicide prevention field, reflections on the definition of lived experience, and the scope of the speaking opportunities available. It is important to that note that the participants had all taken part in lived experience speaking training during the previous six years (from end of the data collection period, being February 2019). Training undertaken had been delivered by a variety of organisations, including Suicide Prevention Australia, Roses in the Ocean, Lifespan/Black Dog Institute, Mates in Construction or via an information manual. Some participants had undertaken more than one form of training. The number of events that participants had participated in ranged from single, isolated speaking opportunities to multiple, regular opportunities (such as speaking, providing content via social media participation, assisting to review programs and policies, committee work) to share their experiences. The participants had experiences of being bereaved by the suicide of a loved one, or via their own attempts. Some had both experiences. An overview of participants can be found in Table 1. 

From an analysis perspective, the in-depth interviews yielded responses that identified complex balances required when “living” lived experience at the same time as participating in activities that required an individual to draw from this experience. This is not a static reflective practice, but a merging of, in some circumstances, professional and personal skills, where complexities regarding the practical and emotional impacts and benefits of lived experience speaking were identified. These two points are important in framing the lens by which these findings are presented. The participants’ experiences of speaking were situated in three ways: those with minimal opportunities for engagement; those with multiple opportunities to utilise their training yet, due to the mechanisms of speaking as a non-evaluated activity, there were varying outcomes; those who wanted to work beyond the scope of simply sharing narratives. These experiences shaped the way in which individual participants were able to reflect on the suicide prevention sector more broadly, along with their role within it. The themes identified from the data are presented in Table 2.

### 3.1. Definitional Challenges and a Lack of Consensus 

As per the interview guide, all participants were asked about their understanding of, and thoughts on, the current definition of lived experience as it is applied in Australia—that is, those who have attempted suicide, carers of people who are, or were previously, suicidal, and those bereaved by suicide. However, for some who had been involved in the field for some time, they were also aware that a previous definition also included people who were affected by suicide in some other way. Constrained by the newer definition which excludes this broader inclusion, participants identified a need for training to reflect some flexibility in order to capture people’s broad range of experience. Flexibility within a definition was required in order to acknowledge that experiences with suicide are not static and based in one event, but change over their life and can be based in myriad events. 

The participants reflected that while the overarching definition of what constitutes lived experience may conform to a general public understanding (such as absolutes like ‘a suicide attempt’ or ‘bereaved by suicide’), it did not accurately reflect their lived experiences which were often far more nuanced. Participants’ views on the definition depended on the participant’s experience of suicide. This was the basis for strongly held views, which were often polarising. Those with mixed experiences of suicide (for example, their own, and exposure to others) were often more likely to support broader definitions, whereas those bereaved by suicide were less likely to do so. Some participants found a limited definition to exclude people who may see themselves as having been impacted by suicide:

*“I don’t agree with the national definition. It’s actually been part of my involvement in lived experience, the challenge with this definition. Most of the committee believed that ‘touched by suicide in some other way,’ was not reflective of lived experience. We can’t judge other people and say that you don’t have a lived experience.”*

(Olivia)

In contrast, others believed a broad definition of lived experience was potentially damaging to individuals, because they felt that not all those with lived experience shared these experiences. Some participants noted that it was difficult for some to understand others points of view, and thus that they were not “representative” of the views of lived experience:

*“There’s no benefit of putting people that have been bereaved [by suicide] with people [who have attempted suicide]. With people who talking about the issues that they face with, [like] you know going to see a psychiatrist, I didn’t see any advantage, I actually walked out.”*

(Jennifer)

When a definition is not shared, such as was uncovered among these participants, a need for greater clarity in both the purpose and role of speakers’ training and the aims of any service that wishes to include lived experience speakers is needed to ensure the right people are involved in experience-matched activities.

### 3.2. Awareness of the Benefits from Lived Experience Participation 

The main motivation that people shared for taking on lived experience role/s was for self-described altruistic reasons. They did not want others to suffer as they had, and by furthering the broader understanding of lived experience they may prevent another’s pain. In learning to tell their stories through the training, participants felt they were also healing as they helped others:

*“I can understand it is difficult for people but in my experience, the more I talk about it, the more natural it becomes and the more accepting I become that it has happened.”*

(Carol)

Broadly, the participants identified that lived experience training was beneficial for them and for suicide prevention broadly, including how being involved and sharing their experiences assisted with their own meaning making. Many described their experience of unintended therapeutic benefit from writing their story during training or being given the space to reflect on the component parts of their story of suicide, within a supportive, facilitated environment provided at the training they had undertaken:

*“Just sharing your experience might just help someone you know… I found the actual training itself was good because it taught me to a point that I probably needed to get through [the impact] and go past as well… I was always very worried about talking to people about it and how they would react and obviously, such a positive [training experience]. I feel I’m in a comfortable place.”*

(Alice)

Altruistic motives were commonly expressed for becoming involved. In this clear example, the simple desire for health care workers to see the suicidal person as more than their current state:

*“That what you see in front of him who’s been a man who’d been very successful, who had held down a very good job, who’d been good fun, absolutely life at the party…everything you know to live for until at 35 he had his first bout of depression. And you know, the end of what you saw when he died was just not the same person and I want…I’d like for my story for people to think…just to think twice.”*

(Jennifer)

While altruism was often a starting point for initially becoming involved, this motivation evolved and matured for those who had been more intensively involved, or involved for longer periods. Furthermore, being engaged in the suicide prevention sector in a meaningful and productive manner led some participants to recognise how speaking out could create a community of people who shared a similar experience, reducing isolation and feeling stigmatised:

*“It makes people realise they’re not alone. That was the one thing that got me going was a lot of people bereaved by suicide, they’re not allowed to talk about it because ‘oh my god, they died by suicide’… They don’t feel that it’s acceptable and you know, when I do these talks just these informal or other talks, people will come up and say thank you. Now I know I’m not alone.”*

(Jessica)

### 3.3. Challenges that Stem from Lived Experience Involvement

While many were positive about their experiences overall, participants shared concerns about times they had felt their suicide-related experience was misunderstood or undervalued. When reflecting on their decisions to step into the role of lived experience representative, participants strongly described needing to be tenacious and resilient. Participants wanted these traits acknowledged, because even those who considered themselves to be ‘fine’ had at times found the reflective practice difficult. This was expressed as occurring at their training, right through to recent events that they had participated in:

*“It’s [training] incredibly cathartic, albeit triggering. I was arrogant enough to think, I’ll be fine and after the first weekend’s training, I was ravaged. I just wept all weekend [be]cause it clearly is very triggering. I think the rawness and the honesty of it.”*

(Amanda)

Significant interactions could make speaking difficult at different times. Some participants identified their first time speaking publicly as ‘nerve-wracking’ because of triggers they had not even anticipated, and not covered in the training, pointing to the difficult task of predicting all situations that may emotionally trigger them:

*“I didn’t know who was going to be there obviously, there was people who had lost loved ones to suicide and the fellow who sat next to me his own mother had attempted suicide and I was doing fine til he said, ‘you know how hard it is for a child to see their mother in hospital after she’s just attempted suicide’ and my kids came to mind and that was a bit of a struggle.”*

(Alice)

Many participants had been exposed to significant and multiple traumas—in relation to their suicide experience as well as from family or origin or external causes. Yet no participants expressed their experience of a trauma-informed approach to training or the activities they participated in. Further, many participants acknowledged that their own personalities did not always lend themselves to self-care; thus, they had to learn how to both identify when they were tired but also how to take care of themselves when reflecting on their suicide experience:

*“When you go back to the toughest part of your life, there’s always a toll and it took a lot of learning on how to put in place strategies to protect myself. I’ll treat myself to a massage or I’ll make sure I have the day off after sharing and things like that. I shared three times in one day [last year] and the third time, I was really exhausted physically and emotionally and so I kind of broke down during the third one. [There’s an] emotional toll there.”*

(David)

### 3.4. Ongoing Care Needs to be a Priority

The bases for decisions on how and why to tell their story and how to be involved in suicide prevention activities changed through experience, whereby participants needed space to conceptualise what lived experience meant to them as well as scope for their story to keep shifting as time went on. Participants noted that initial training needed to be more than helping them to develop skills in storytelling, but to engage with and spend time understanding the layers of experience they were prepared to share publicly. There was significant breadth in how this was expressed, with some experiences that were very positive and immediately opened up opportunities:

*“My first storytelling was to my friends. So, I had a barbeque at my house. I invited a special group of people together and I told them my story and for some of them that was the first time they were fully understanding the whole thing. [They] quietly told me that they had attempted themselves and they’ve never shared that with anybody.”*

(Adam)

Other experiences were described as emotionally draining given reactions to their shared experience were not always positive or opened up stories from other people that they may not have had the skills to manage:

*“So, it’s not so much even really about making sure that you’re comfortable with things that you say, it’s about making sure that you’re comfortable with the way that people are going to take it.”*

(Olivia)

Others noted that not everyone who completed lived experience training may be ready to become a lived experience representative. Yet, there is no apparent assessment of who is ready, willing and able to speak in the community. There did not appear to be acknowledgement of how experiences may change over time, and who might need additional training, debriefing or support. Following the initial training, it appeared that ongoing development was left up to individuals:

*“I think I was comfortable in why I wanted to tell mine [story]. There was a lot of how to do it and I think there was just a general acceptance that people could self-monitor.”*

(Ruth)

From a safety perspective, many of the participants noted limited engagement post-training from those with whom they had trained. No participants spoke about longer-term professional development, nor how the lived experience role was changing rapidly and how this might be reflected in established training. There is currently no Australian register of individuals who are involved in representing lived experience of suicide, and no way to determine who among those in these roles have had any training pre-engagement. Some participants expressed a desire for ongoing support, particularly in the early days of engagement in lived experience activities. One participant suggested three-month follow ups with each person that had completed training, where space was created for the person to reflect on their motivations:

*“Training [is] not about doing public speaking necessarily, but having a space to find your story. You get exposed to being able to actually talk to people who’ve had a potentially opposite experience to you in terms of an attempt. You start putting jigsaw pieces together that you can’t get the answer from your loved one, “cause they’re not here.”*

(Amy)

When asked about how individuals network with others engaged in these activities, no participant was aware of a formalised way in which to do this, although those who were involved in greater depth had informal networks. Further, the nature of some engagements meant that there was no opportunity for feedback from their involvement. In community events, participants talked of often leaving events or conferences soon after speaking. The outcome of this is people are left in potentially vulnerable states with no follow up:

*“After speaking, people who are inviting you to speak, [should] just offer some way or just let us know if it becomes too much or if you need to take a break we can assist you in that because there is that responsibility of care.”*

(Sasha)

Engagement between activity organisers and those offering their lived experience to the activity, was haphazard among these participants. Some reflections emphasised that inclusion in activities was positive for some, there were concerning experiences where individuals were left in vulnerable states after engagement. With no clear follow-up protocol to support them through that vulnerability.

### 3.5. The Practical and Emotional Labour of Speaking

There were additional emotional impacts that stem from these activities, and resultant practical implications can be significant. Participants noted that there is no one to assist speakers who have been trained, which limits the opportunity to match speakers to events. There were common experiences of haphazard arrangement of event organisers including how they were located to be the provider of lived experience or where they were invited to be lived experience representatives around a policy or project table. From the experiences of these participants, it appears that individuals are often matched to events through availability rather than matched expectations related to the purpose of the engagement or via word of mouth. This resulted in participants often being unaware of what the organiser wanted from them, and thus they were often ill-prepared for the event. However, while almost all participants spoke about wanting to tailor their story to the audience where possible, many viewed the ambiguity of their plans as just part of being involved in lived experience:

*“A lot of it is just through word of mouth. I’ve had you know friends different [places] say, ‘hey look we’re doing a mental health day. Can you come and present?’ I have had other groups who have seen me on TV and has sought me out and reached out to me and things like that.”*

(Peter)

For others, the decision of what to share and when was much more fluid in terms of what they defined as an engagement:

*“I’m on [a] ridiculous number of advisory committees and you have to bring your lived experience for that table, if it’s relevant. And if not, then I bring the perspective of others that we’ve learned from over the years. [I] try and bring their voice to the table if they’re not there. I talk about my experience a lot.”*

(Amy)

**Figure 1:**
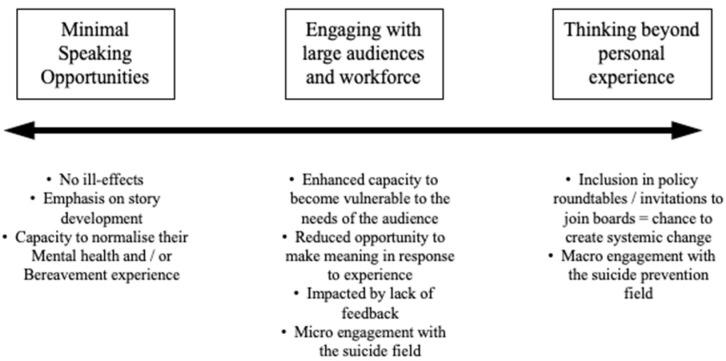
A developmental continuum: identification of experiences.

Learning how to use a variety of people’s experiences in this way was rare among participants, which may relate to the novelty of this activity in the field of suicide prevention, or for some it is more difficult to move into a position of using the broader term of lived experience to contribute beyond personal experience alone. The depth of experience and breadth of activities individuals had participated in contributed to this ability to share broader perspectives, and perhaps fulfils a different role than those for whom engagement in this field is share their experience to reduce stigma and increase help seeking through community events. Analysis of these themes results in the presentation of a continuum of involvement, as presented in Figure 1. This reflects the manner in which participants stated that they chose to take part in lived experience research and their experience of doing so. While reflecting on earlier experiences, those who were more able to see their contribution as being beyond their own personal involvement tended to have spent more time or had been more active in their activities in the suicide prevention sector.

Sharing experiences of suicide reveals deep personal vulnerability and includes intimate reflections on some of the most challenging situations a person can be faced with. How people viewed these changed, and yet once shared, their intimate experiences and vulnerability became public. Better assessing why people become involved, what kind of involvement they wish to have (noting this may change), and how their own experience will be used remains poorly understood. While in no way representative, our sample was dominated by those who had fewer experiences, with only a few who were more broadly involved in the suicide prevention sector. This could be a result of the infancy of the field.

## 4. Discussion

This paper explores the narratives of twenty participants who self-identified as being involved in lived experience activities in Australia. Their sharing included opportunities to present to mental health panels, the media, online, during conferences and in submissions seeking the input of those with lived experience of suicidal ideation or those bereaved by suicide. The broad definition of lived experience in Australia encompasses the stories of those bereaved by suicide as well as those who have cared for someone who has made a suicide attempt, and those who have attempted to end their lives. Often these experiences are overlapping. Lived experience involvement has a variety of purposes; for example, the literature identifies that lived experience can both demonstrate why an intervention may or may not be effective or appropriate, as well as how that intervention works in everyday life [7]. However, in this sample, the most common experience was sharing a lived experience of suicide in a public forum with the perceived expectation that this will better inform audiences of what suicide is, and how suicide is experienced. How people managed their well-being was up to the individual, with no structure in place. While this might be sufficient for some, for others, this will not meet their needs. This could be especially exacerbated if an individual had had minimal opportunities to tell their story and managing the reactions of others could be painful each time. It is well established in the literature that supporting people through vulnerability can be protective. The driver for continued engagement in sharing lived experience narratives was expressed as needing to de-stigmatise what were commonly stigmatised issues. Yet, this comes at a personal cost to those who are championing new recognition of a previously taboo topic see reference [10].

There is no way of determining how people were able to measure the impact (or what impact they might measure) through their lived experience participation. Some were focused on the possibility that someone in the audience would take something from their story and that was perceived as satisfactory. Others were less sure. Yet others perceived impact in changes at a macro level rather than the micro. Acknowledgement is urgently needed that people come to these activities with different needs, strengths and attributes, as well as different support needs. It is also important to identify that impacts may be sudden and distressing, whereas others may require support over time depending on how they continue to report their experience of sharing [14,19]. Continued involvement in lived experience speaking might be detrimental to people’s mental health. The research is unclear as to whether this detriment, in the longer term, may force people away from the consumer workforce. Without measures that capture the impact of these activities on people and proactive support systems, there is significant vulnerability in this rapidly developing field. Discussions regarding the use of feedback questionnaires (pre- and post-event) to measure how the audiences’ knowledge of and attitudes to suicide has changed (given a majority of this samples’ experience is talking in a public forum with the expectation it will increase the audiences’ knowledge was noted) may be an important inclusion not only for event organisers but capacity for speakers to reflect on the impact of their work, as a measure of impact on themselves.

We are now at an academic impasse, where the lived experience movement in the Australian suicide prevention sector requires deeper understanding about what inclusion of lived experience is and how ‘living’ the lived experience can enhance suicide prevention activities. In thinking beyond our current activity based focus, in terms of how this can sway public sentiment, impact policy and intervention development, ‘lived experience’ needs to be unpacked. This study confirms that conceptualising what lived experience means to individuals, and how people share this in their activities, is important, particularly as the field of lived experience gains momentum. Who is viewed as being able to authentically lend their voice and experience to inform others from a lived experience perspective is intrinsically important both to those sharing and those receiving insights. Given that the purpose of the inclusion of lived experience of suicide to the field of suicide prevention is to draw in the first-hand accounts of those who know suicide best, inclusion and exclusion appears to take the focus off this premise. Rather than focusing on definition alone, it is vital for the suicide prevention sector to better understand the role of suicide in people’s lives, and how those with lived experience can most valuably help while being best supported, to enable more appropriate activities for preventing suicide and suicide-related harm.

The participants’ narrated experiences informed the results and the conceptualisation of the Continuum of Lived Experience presented in Figure 1. How participants experienced training and speaking activities, how they lived their ongoing lived experience, shapes the national discourse on suicide prevention, and this should be respected. It also became clear that the opportunities to complete training and then go on to do intermittent or one-off speaking engagements offered a chance for people to make meaning from their previous experiences and to understand their own story better. However, inclusion in policy development or on committees or roundtables—where speaking was prolific, and word of mouth spread—created challenges in the practical and emotional costs of speaking. Those who appeared to be impacted heavily by their speaking went on to work more specifically in the macro space of lived experience speaking. Alternatively, some walked away from speaking because of the disorganised space where inclusion of lived experience has been created. Assessment for whom different activities are appropriate, and with what supports in place, is urgently needed

The participant narratives reflected a mix between altruism that involves meaning making alongside the role of lived experience speaking as a pseudo public service or public health promotion campaign, oscillating between audience engagement and moving beyond their own experiences. Speaking provides the opportunity to reflect on personal loss, or their own mental health journey and a way to succinctly collect their experiences as they develop a narrative of their lived experience. Of those who agreed to share their experiences of speaking training, the underlying identity as ‘speaker’ was prevalent—this was the word people often used to describe themselves to others. Further, while the experience of speaking prompted desire to continue to share their narratives in many, there was sometimes little way to predict positive and detrimental outcomes. Emotional reactions to experiences could be both. Yet, the discounting of being emotional when talking about suicide raises attention to a tension in the field.

As new research is undertaken on lived experience, including this study, we are able as a community to not only understand more about the breadth of experience of suicide, but also be able to better include more people in the field. The potential to capture a wider breadth of these lived experience narratives, and analyse them within rigorous and participatory frameworks, places qualitative research in a unique position to inform future suicide prevention initiatives in practical ways. This study raises questions regarding who may be deemed as having a valuable message and how those deemed less valuable understand their experience. Several immediate recommendations can be made. First, a screening process is required to understand the reasons people get involved in becoming a lived experience representative. This will allow for training to be tailored to individuals needs, which can in turn be used as a measure of success for that person to meet their desired outcomes from becoming involved. Caution should be used to ensure that any screening is not paternalistic. Second, a comprehensive framework for debriefing and support is urgently required, including assessment of peer versus professional mentoring. Third, a mechanism for networking with others participating in these activities can provide peer support as an additional way to support those doing this important work.

## 5. Limitations

This qualitative project included a small sample to commence a broad understanding of how people experience sharing their personal experiences to prevent suicide. Thus, these findings are limited and it is likely that there will be similar and different experiences among others. Further, such findings may not be generalisable to a wider population. In addition, given the novel approach to this project, and the emphasis on seeking understanding of inclusion of those with lived experience, in a style of work that is characterised by only seeking inclusion of lived experience voices, it remains difficult to identify common themes with other research projects given the dearth of research in this area of suicide prevention. However, despite these limitations, this study was able to capture a wide variety of lived experiences of suicide, and different training and speaking experiences, which demonstrate the difficulty in binding this population into too narrow a representation.

## 6. Conclusion

Authentic inclusion of lived experience in the suicide prevention space is precarious work, because it seeks more than valuing the existence of lived experience but to create a supportive environment where the true work of honouring these narratives and shaping the mental health system in response to it, can take place. Not having caveats around what experience is required for certain audiences means that inclusion of lived experience knowledge simply does not always fit the context that it is included within.

Despite the complexity of responses, it is clear that training can never meet all the needs of individuals who seek out support to tell their own story of suicidality. From an individual perspective, the participants noted that there is a breadth of experience of speakers in terms of the amount of time they have been actively engaged in speaking activities; however, there is a dearth of research about the impact of what occurs for people who have been talking for longer periods about their experience, especially in terms of how they manage self-care and what triggers the need for breaks or to step away from the suicide prevention sector.

Overall, the participants described variable post-training experiences which identified that people’s access to speaking opportunities appeared limited in direction and planning. Participants described screening processes as to who can attend speaker training that appear paternalistic at times, with judgements made as to whether a person is ‘well enough’ to speak, rather than using screening tools to identify the wellness of an individual or their goal as to what they hope to achieve by sharing their insights.

Suicide is a complex and emotional issue that does require people to be challenged and be challenging. The community is often presented in person or online with the voices of those with lived experience, yet we have focused little attention and funding on the implications of being behind that voice. What we know from this study is that for those who were working full time in the suicide prevention field, overexposure to suicide stories could be problematic, or could become so, after hearing too many stories from others. A national strategy that seeks to better support the authentic inclusion of lived experience speakers in all areas of suicide prevention, which identifies how living the lived experience has capacity to shape the way the sector responds to those with future needs, is required. Further research is desperately needed to better understand the needs of those involved in this work and how best to support them.

## Figures and Tables

**Table 1:** Participant information. The participants had experiences of being bereaved by the suicide of a loved one, or via their own attempts. Some had both experiences. Legend = Male (M), Female (F), x = experience defined by participant, LEX = Lived Experience.

Pseudonym	Gender	Bereaved by Suicide	Own Suicide Attempt	Both Bereaved by Suicide and Own Lived Experience of Attempting	Length of Time Engaged in LEX Activities
Ruth	F			x	1.5 years
Christian	M	x			2 years
Sasha	F		x		2 years
Aalia	F		x		3 years
Carol	F	x			3 years
Mary	F	x			3 years
Rebecca	F	x			3.5 years
Alice	F		x		4 years
Adam	M	x			4 years
Elizabeth	F			x	4–5 years
Peter	M	x			5 years
Jennifer	F	x			6 months
Jessica	F	x			6 months
David	M				6 years
Andy	M			x	6 years
Amanda	F		x		6 years
Olivia	F		x		7 years
Amy	F	x			7 years
Eva	F		x		8 years
Ray	M			x	10+ years

**Table 2:** Themes identified, lived experience (LEX) engagement in suicide prevention and impacts of speaking.

Theme	Thematic Content
*Definitional challenges and a lack of consensus*	The Australian definition of lived experience (those who have attempted suicide, carers of people who are, or were previously, suicidal, and those bereaved by suicide) identified a disconnect between what is ‘lived experience’ due to nuances shared by participants about their lives and exposure to suicide
	Experiences of speaking reflected that a broad definition was not always encouraged, especially if bereaved
	Differing views during speaking engagements needed to be better matched to audiences, rather than generic referral of LEX speakers
*Awareness of the benefits from lived experience participation*	Involvement for altruistic reasons, to minimise suffering in others
	Training provided an opportunity for meaning making
	Longer-term engagement highlighted that the community needed to better respond to lived experience
*Challenges that stem from lived experience involvement*	Sharing LEX and responding to audiences led to some feeling not understood/ undervalued
	Speaking could induce nerves or trigger reminders of loss or trauma
	Post-speaking triggers were sometimes poorly managed by the organisations inviting LEX speakers
	Lack of trauma-informed approach to training or the activities speakers participated in. This needs to be addressed
*Ongoing care needs to be a priority*	Lived experience is ongoing; the scope of a person’s story will shift, and not remain static
	Speaking could be “emotionally draining”. Speakers are not equipped to respond to the audience
	No assessment of who is ready, willing and able to speak
	Post-training support not provided
	LEX has informal networks for some; however, this needs to be formalised
*The practical and emotional labour of speaking*	Repetitive episodes of vulnerability can take their toll on people, vicariously and directly
	Expertise is often focussed solely on LEX and not alternate skills speakers possessed
	Assessing why people engage in speaker training, their involvement and experience broadly requires future research

## Appendix A. Interview Guide

Authentic inclusion of lived experience—a pilot study

Interview script, consent and questions

Interview no:
Date:
Time:
Interviewer:

Thank you for agreeing to speak with me today about your experience of sharing your lived experience of suicide as a trained speaker. We will want to ask you some questions about your decisions to share your story and the positives, barriers and challenges that might come from doing that. The interview will take approximately 45 to 60 min. If you feel that you would rather not go on with the interview that is fine too. At the end of the interview, we will discuss the supports you have available to you.

Now I just need to confirm some information about you, and I’m going to turn on the tape recorder. This will help us to accurately record your story, but all this information will remain completely confidential. Is that OK?

First, I need to ask you some questions to confirm that you consent to participating. Remember, even after you’ve answered these questions, you can withdraw your consent at any time during the interview. The consent questions are:

Consent Question	Yes	No
Have you read the information contained in the information sheet for participants and had any questions answered to your satisfaction?		
Do you agree to participate in this activity, with the understanding that you may withdraw at any time?		
Do you agree that research data gathered for the study may be published using a pseudonym?		
Do you agree that you may be quoted using a pseudonym?		
Do you agree to the interview having your audio recorded and transcribed?		
Are you aged 18 and above?		
(If answered No to any of these—clarify and/or discontinue interview).		

To begin, I’d like to ask you some questions about you, so we can get an understanding of who is participating in this research:

- Can you please tell me what year you were born?
- How would you describe your gender?
- Were you born in Australia? If not, where were you born?
- What language do you speak at home?
- Are you of Aboriginal and Torres Strait Islander descent? Aboriginal/Torres Strait Islander/Aboriginal and Torres Strait Islander?

Now I’ll be asking you some questions about your experience of sharing lived experience reflections. We are interested to know:

1. Seek clarification on what experience of suicide they have experienced.
  - Died by suicide? Attempted suicide? Cared for someone suicidal?
  - What was your relationship to them, time since last attempt (if multiple) or death
2. If only attempt survivor go to Q3. What is the experience that motivated you to become involved in sharing your experience (if multiple, ask for most impactful, only if they have known someone who has died or attempted, not self-attempts)
  - Thinking about the effect of the person’s death/attempt on your life, please indicate the rating that best describes your experience by selecting the most relevant descriptions.
    The suicide death had little effect on my life.
    The suicide death had somewhat of an effect on me but did not disrupt my life.
    The suicide death disrupted my life for a short time.
    The suicide death disrupted my life in a significant or devastating way, but I no longer feel that way.
    The suicide death had a significant or devastating effect on me that I still feel.
  - Continuing to think about the person whose suicide attempt/death affected you most, how close would you describe your relationship with this person?
    Not close  A bit close  Moderately close  Close  Very close
  - During the six months prior to their attempt, on average, how often did you have any contact with them (e.g., in person, phone or by email etc).
    Daily  Every few days  Weekly  Every few weeks  Monthly  Infrequently
3. What does lived experience mean to you?
4. What roles have you undertaken sharing your lived experience?
5. In what forums have you shared your story?
6. How do you feel your story is ‘heard’? How do you know the audience hears your story?
7. Have you experienced negativity or exhaustion from sharing your story?
8. Do you think you will stop or take a break from doing this? When/Why?
9. Do you ever compare your story to others who share there’s? How have you managed this?
10. What have you needed in relation to support in deciding how often you share/don’t share your story? How do your family and/or friends react to you being a speaker on lived experience?
11. Do you receive supervision or support from others (professional? Peer?) in relation to sharing your story of lived experience?
12. Have you experienced any conflict between your professional role and your personal lived experiences?

Experience of training:

13. Have you received formal or informal training?
14. From whom?
15. When?
16. What did you think of this training?
  - What was covered in your training?
  - What were the gaps?
  - Who organises your speaking engagements?
  - Managing the practicalities of speaking, including the costs associated with speaking?
  - How do you manage informal conversations with the audience before/after speaking engagements?
17. What has been your experience of ongoing support from the organisations you speak to or on behalf of?

Now thinking more broadly about the role of lived experience speakers:

18. What do you think motivates people in and out of the ‘job’ of lived experience speaking?
19. What helps/hinders involvement in lived experience?
20. Why do you think a variety of perspectives of lived experience is important?
21. *In Australia we use a broad definition of lived experience to include those who are bereaved, those who have attempted, and those who have supported someone who has attempted or died by suicide.* What are your thoughts on an inclusive definition? Do you think there is a place to separate these groups?
22. Do you think that those who are bereaved are assisted by hearing about those who have survived a suicide attempt and vice versa? In what ways?
23. What influence do you think sharing your story has on others? What examples do you have of this influence?
24. Is there anything else we haven’t asked that you wish to share?

Just to finish off, what was it that motivated you to reach out to us to be interviewed for this research?

What has been your experience of participating in this research?

Thank you so much for talking time to talk to us. I hope that this research can help others who make the decision to share their lived experience. Your insights are really valuable and it’s so important that stories like yours help us to understand how to help others.

How are you feeling now that the interview is over? Can you think about an activity that you can do today that will be positive for your mental health? For example, it may be going for a walk, having a coffee with a friend…

You might find that talking to me has raised some concerns or thoughts for you and talking about it with someone could help. Would you like the research team to give you a call in a couple of days?

(If yes) What is the best time and contact number to reach you on?

Do you have formal support that you can contact if you are concerned about the content of this interview, for example, the organisation you undertook your speaker training with, a counsellor or other health professional?

Now that we have completed this interview, we will transcribe the audio file into a written document. You have the option to see this document to verify that the transcript is correct or change anything that you would like changed. Would you like to see the transcript before we analyse the data?

(If yes) Would you like me to email or mail that to you? What is the best address to send it you?

We sent out some information and referrals to you when you signed up. Do you still have those? If not, I can get you another copy of that information.

Do you feel all right to end the call now? Thanks again for participating.
